# Molecular Signaling Pathways in Wound-Induced Hair-Follicle Neogenesis

**DOI:** 10.3390/cells14060440

**Published:** 2025-03-16

**Authors:** Soung-Hoon Lee

**Affiliations:** Department of Anatomy, Yonsei University College of Medicine, Seoul 03722, Republic of Korea; greateondal84@yuhs.ac

**Keywords:** wound-induced hair-follicle neogenesis, hair-follicle regeneration, molecular signaling pathways, Wnt signaling, drug development

## Abstract

Wound-induced hair-follicle neogenesis (WIHN) is the phenomenon of regenerating new hair follicles from wounds in mammals. The WIHN involves both developmental and adult wound-healing processes. Moreover, the WIHN is regulated by a variety of factors, particularly multiple molecular signaling pathways produced in several types of cells. Here, the role of multiple signaling in different types of cells in WIHN is comprehensively described. Furthermore, the lack of dermal γδ T cells in the human scalp has hindered the clinical application of WIHN, but the development of drugs such as Wnt signaling activators is increasing the effectiveness of WIHN in humans. Overall, understanding the underlying mechanisms that regulate WIHN may help treat skin diseases, including alopecia.

## 1. Wound-Induced Hair-Follicle Neogenesis (WIHN) as a Hair-Follicle Regeneration Phenomenon

In mammals, some regenerative organs, such as the skin and alimentary canal, maintain tissue function using tissue-resident stem cells [1]. Skin regeneration, especially, has been studied for a long time to treat various incurable skin diseases [2].

In 2007, a phenomenon of hair-follicle regeneration called wound-induced hair-follicle neogenesis (WIHN) was first reported [3]. The WIHN is a phenomenon in which new hair follicles regenerate from wounds in mammals, and this phenomenon has been observed in mice, rats, rabbits, sheep, and humans [3,4,5,6]. The nascent hair follicle formed by wounding establishes a stem-cell population and ultimately becomes a mature hair follicle by expressing key markers of hair-follicle differentiation [3].

This discovery overturned previous ideas that hair-follicle loss in adults was permanent and that mammalian hair follicles formed only during development [3]. It has been demonstrated that wounding can induce an embryonic phenotype even in adult skin [3]. However, a recent report has shown that embryonic hair-follicle development resurrects after wounding in adult skin but may not necessarily utilize the same molecular signaling used in embryonic development [7]. In other words, the WIHN includes both developmental and adult wound-healing processes [7]. Overall, the WIHN is a hair-follicle regeneration phenomenon that occurs in adults, and subsequent studies have been conducted to find factors that can activate this phenomenon.

## 2. The Three Major Factors Regulating the WIHN

The WIHN is a regenerative phenomenon that can be exploited in the treatment of alopecia and is regulated by various factors [3,8]. In particular, the WIHN is mainly regulated by the following three factors.

**Figure 1:**
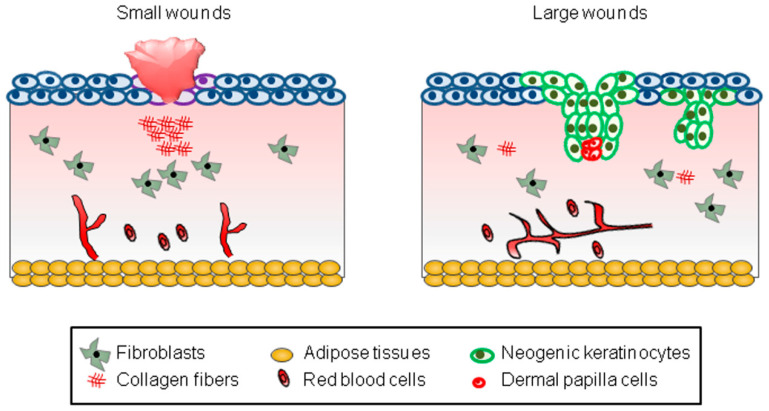
A schematic model representing WIHN. The schematic pictures show scar formation and WIHN in small and large wounds, respectively. The text in the square box below describes each cell type.

First of all, the WIHN occurs depending on the size of the wound created [3,9,10]. Most small wounds (wounds less than 0.25 cm^2^ in mice) fail to cause the WIHN, leaving only a scar (Figure 1). On the other hand, most large wounds (wounds larger than 2.25 cm^2^ in mice) successfully induce the WIHN (Figure 1). However, since it is clinically impossible to create large wounds on the human scalp, efforts should be made to induce the WIHN even in small wounds.

Second, the WIHN is closely related to the age of experimental animals [3]. In young mice, the WIHN occurs frequently (wounds larger than 1 cm^2^ in 3-week-old mice), but with age, the WIHN becomes limited to large wounds (wounds larger than 2.25 cm^2^ in 3-week-old mice) [3]. This phenomenon is consistent with the decline in regenerative capacity with age in most tissues in mammals [11]. However, considering that most alopecia occurs in adults, it is important to induce the WIHN in adults.

Finally, multiple molecular signaling pathways are involved in the regulation of WIHN. The importance of these molecular signaling pathways in the WIHN has been proven by previous studies using multiple transgenic mice [3,8]. The roles of multiple signaling pathways in the WIHN will be discussed in detail in Section 3.

## 3. The Multiple Signaling Pathways Involved in the WIHN

**Figure 2:**
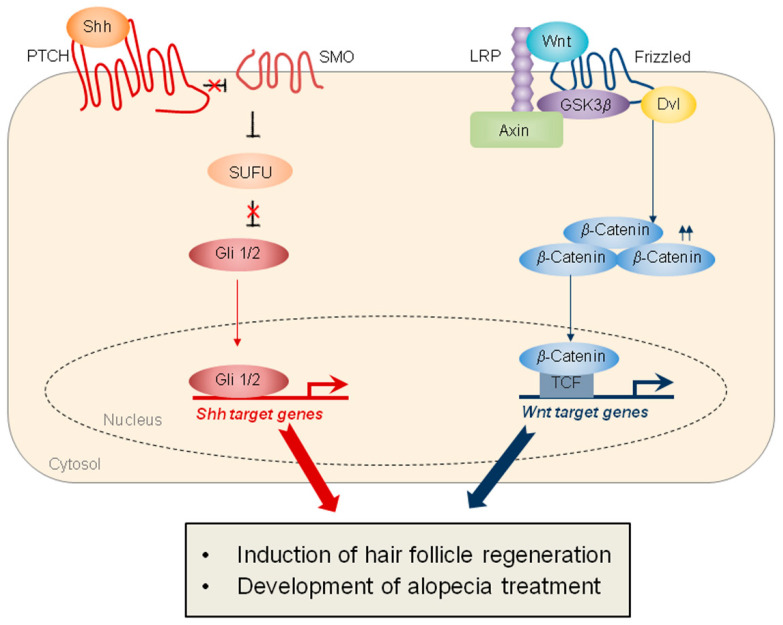
Schematics representing SHH and Wnt signaling, which play important roles in hair-follicle regeneration. (**Left**) SHH signaling is mediated by the Patched (PTCH), which inhibits SMO. The activation of SMO promotes the release of Gli 1/2 proteins from the suppressor of fused (SUFU), thereby inducing the transcription of SHH target genes. (**Right**) Wnt signaling is mediated by the Fizzled and the lipoprotein receptor-related protein (LRP). In response to Wnt, Wnt receptors recruit Axin and GSK3β, leading to the stabilization of β-catenin. The nuclear β-catenin binds to the T-cell factor (TCF) transcription factor, resulting in the transcription of Wnt target genes. The target proteins activated by both signaling pathways induce hair-follicle regeneration and contribute to the development of treatments for alopecia.

Several signaling pathways are involved in the WIHN, the two main pathways being Wnt signaling and sonic hedgehog (SHH) signaling (Figure 2). Specifically, the epithelial overexpression of Wnt7a, a Wnt ligand, significantly induces the WIHN, while the epithelial overexpression of Dickkopf-1 (DKK-1), an inhibitor of Wnt signaling, markedly inhibits WIHN [3]. Moreover, epithelial SHH overexpression or dermal smoothened (SMO) activation induces extensive WIHN in mouse wounds [8]. Although the crosstalk between Wnt signaling and SHH signaling has been studied in other types of tissue regeneration [12], the cooperation of these two pathways in hair-follicle regeneration is not fully understood (Figure 2). Ultimately, the coordinated regulation of these two major pathways will be key to inducing regenerative healing and hair-follicle regeneration (Table 1).

In addition to these two major signaling pathways, various molecular signaling pathways are involved in hair-follicle regeneration (Table 1). For instance, the activation of insulin-like growth factor 1 (IGF1) signaling promotes the WIHN through the contribution of epidermal growth-factor receptor (EGFR)-positive mesenchymal cells [13]. The identification of EGFR-positive cells within dermal fibroblasts enables the potential utilization of this subpopulation in hair-follicle regeneration (Table 1). Bone morphogenetic protein (BMP) signaling has recently been shown to be essential for hair-follicle regeneration, contrary to traditional notions of its influence on hair-follicle cycling [14]. Germline or conditional knockout of *Msx2*, a bona fide target of BMP signaling, revealed delayed wound healing and the significant inhibition of WIHN in big wounds, indicating that BMP signaling is essential for hair-follicle regeneration (Table 1). Hypoxia-inducible factor 1 alpha (HIF-1α) signaling also promotes the WIHN by stimulating glutamine metabolism [15]. The close relationship of HIF-1α signaling with interleukin-1β (IL-1β), metabolic, and regenerative signaling suggests the significance of pro-regenerative metabolic programs in human regeneration (Table 1). In-depth research on these various molecular signaling pathways has provided an opportunity to further determine the relationship between components within these signaling pathways and hair-follicle regeneration.

Indeed, various components within molecular signaling pathways play key roles in hair-follicle regeneration. CXXC-type zinc finger protein 5 (CXXC5) has been identified as a negative regulator of Wnt signaling, and this protein inhibits Wnt signaling and WIHN through interaction with Dishevelled (Dvl) [16,17,18]. Elucidating the function of negative regulators of Wnt signaling, such as CXXC5 and DKK-1, has opened the way to safely regulate Wnt signaling critical for hair-follicle regeneration. The activation of pyruvate kinase M2 (PKM2), one of the key enzymes of tyrosine kinase signaling, also promotes the WIHN through crosstalk with Wnt signaling [19]. Furthermore, OVO homolog-like 1 (OVOL-1), the transcriptional target of Wnt signaling, is closely related to hair-follicle neogenesis [20]. The discovery of molecular targets to induce hair-follicle regeneration has led to the development of drugs to treat hair loss, which will be discussed in Section 7.

## 4. Role of Multiple Signaling Pathways in Stem Cells During the WIHN

Several types of cells are involved in wound healing and WIHN in the wounded skin [21,22]. Among them, stem cells within the skin play a vital role in skin homeostasis, wound healing, and hair regeneration [21]. Among them, hair-follicle stem cells (HFSCs) and melanocyte stem cells (McSCs) play important roles in maintaining the hair growth cycle and regulating hair pigmentation, respectively [23]. HFSCs and McSCs coordinately regulate pigmented hair regeneration, and HFSCs provide a functional niche for McSCs [23,24].

HFSCs play an important role in inducing hair-follicle regeneration [25]. However, the lineage tracing of keratin 15-positive stem cells in an earlier study demonstrated that keratin 15-positive stem cells make only a minimal contribution to WIHN, indicating a major contribution to WIHN from non-hair-follicle bulge cells [3]. In contrast, Lgr5-positive hair-follicle stem cells were found in almost half of hair follicles regenerated from wounds through lineage studies, and studies using transgenic mice showed that Lgr5-positive hair-follicle stem cells significantly contribute to the WIHN [25]. Overall, it can be seen that the activation of specific populations of HFSCs is important for inducing the WIHN.

McSCs are critical for pigmented hair-follicle regeneration [26,27,28,29]. McSCs migrate from the hair-follicle niche to the epidermis after wounding and contribute to the generation of unpigmented hairs in the wounds [26]. However, because the McSCs of melanocortin 1 receptor (Mc1r)-mutant mice show defects in migrating from the hair-follicle niche to the epidermis, pigmented hair is generated in these mice after wounding [26]. The overexpression of Endothelin-1, which is involved in melanocyte homeostasis, promotes the migration of McSCs and the generation of epidermal melanocytes after wounding [27]. The activation of Wnt signaling also promotes the generation of epidermal melanocytes following wounding [28]. Moreover, overexpression of the melanocyte stimulatory factor Kitl in mice led to pigmented hair regeneration after injury [29]. Collectively, the regulation of multiple signaling pathways within HFSCs and McSCs can induce pigmented hair-follicle regeneration in adults after wounding.

## 5. The Role of Multiple Signaling Pathways in Immune Cells During the WIHN

Various types of immune cells and signaling pathways activated by these cells are involved in wound healing and WIHN (Table 2). For example, γδ T cells, characterized by T-cell receptors consisting of γ and δ chains, reside in the epidermis and dermis of the skin and secrete various growth factors associated with wound healing and hair-follicle regeneration [30]. Fibroblast growth factor 9 (FGF9), secreted by dermal γδ T cells, promotes the WIHN by activating Wnt signaling [30]. Toll-like receptor 9 activation in γδ T cells also induces the WIHN by up-regulating the expression of amphiregulin (AREG) [31]. Tumor necrosis factor-α (TNF-α) secreted by macrophages also induces the WIHN by activating AKT/β-catenin signaling [25]. Moreover, macrophage-regulated CX3C motif chemokine receptor 1 (CX3CR1) and transforming growth factor-β1 (TGF-β1) signaling play an important role in inducing hair-follicle regeneration [32]. Recent studies on the roles of immune cells in WIHN indicate that various signaling pathways secreted by cutaneous immune cells are important in the regulation of hair-follicle regeneration.

Additionally, non-immune cells, including keratinocytes and neuronal cells, produce cytokines, thereby regulating the WIHN (Table 2). The binding of Toll-like receptor 3 (TLR3), expressed in keratinocytes of injured tissues, and its ligand, double-stranded RNA (dsRNA), induces the WIHN by up-regulating interleukin-6 (IL-6) expression [33]. The self-noncoding dsRNA can also induce retinoic acid (RA) synthesis, which is involved in promoting the WIHN, through up-regulated IL-6 expression either by wounding in mice or by a rejuvenation laser in humans [34]. A recent study has demonstrated that skin-resident bacteria induce IL-1β production in keratinocytes via HIF-1α signaling, leading to the promotion of WIHN [15]. Moreover, interleukin-36α (IL-36α) expressed in keratinocytes induces the WIHN by activating the IL-6/signal transducer and activator of transcription 3 (STAT3) pathway [35]. Overall, the association of WIHN with the immune cells suggests that WIHN and wound healing involving immune cells may be clinically targeted simultaneously.

## 6. The Role of Multiple Signaling Pathways in Dermal Cells During the WIHN

Recent studies have revealed that dermal cells also play an important role in hair-follicle regeneration [8,36]. For example, the activation of dermal SHH signaling induces extensive WIHN in wounds [8]. Moreover, the epidermal–dermal interactions are crucial for both hair-follicle development and regeneration [37,38]. Importantly, various types of cells in the dermis are involved in hair-follicle regeneration [8,22].

The transition from fibroblasts to myofibroblasts is a key process in wound healing [39]. The myofibroblasts play a key role in wound healing, but their excessive activity can lead to scar formation [40]. Interestingly, recent findings have demonstrated that neogenic follicles can transform myofibroblasts into adipocytes following injury in humans and mice [22]. In particular, neogenic follicles regenerated from the wound activate BMP signaling and induce adipocyte transcription factors such as peroxisome proliferator-activated receptor γ (PPARγ) [22]. Therefore, the overexpression of the BMP antagonist Noggin in hair follicles or the deletion of the BMP receptor in myofibroblasts inhibited the formation of dermal adipocytes in the wounds [22]. Collectively, the regulation of dermal adipocytes through the modulation of BMP signaling may simultaneously inhibit scar formation and induce the WIHN.

## 7. Development of Drugs to Improve the WIHN

Although there have been attempts to apply the WIHN clinically, they have been relatively ineffective due to the lack of dermal γδ T cells in humans [30]. Therefore, the activation of molecular signaling pathways such as Fgf9 and Wnt signaling, regulated by γδ T cells, was required to induce the WIHN in humans [30]. As described earlier, basic research on the molecular signaling pathways that regulate the WIHN has continued for over a decade, followed by applied research to activate these signaling pathways.

Wnt signaling is an attractive target for drug development to induce hair-follicle regeneration (Table 3). Valproic acid (VPA) activates the Wnt signaling and induces the WIHN by inhibiting glycogen synthase kinase-3β (GSK-3β) [41]. 4-phenyl butyric acid (PBA), a derivative of VPA, also induces hair-follicle regeneration by activating the Wnt signaling [41]. Lithium chloride (LiCl), another GSK-3β inhibitor, promotes the pigmented hair regeneration in wounds [29]. The administration of recombinant IL-6, a target protein of Wnt signaling, also significantly induces the WIHN [33].

Many substances activating Wnt signaling have been tested preclinically to increase the effectiveness of WIHN, but most of them have not been tested clinically because of the worrisome issues of this signaling in relation to cancer [42]. To resolve the issues of Wnt signaling, the ways to safely target this signaling were explored, and one of them was to indirectly activate Wnt signaling by inhibiting the function of negative regulators of Wnt signaling, such as CXXC5 [17,42]. Protein transduction domain–Dishevelled binding motif (PTD-DBM), a peptide that inhibits CXXC5-Dvl interaction, induces the WIHN by activating Wnt signaling [17]. KY19382, a mimetic small molecular compound of PTD-DBM, also significantly induces WIHN by activating Wnt signaling [43].

The pharmacological activation of Wnt signaling through crosstalk with other signaling is also known to markedly increase the WIHN [19,44]. TEPP-46, a selective activator of PKM2, promotes the WIHN, and its combination treatment with Wnt signaling activators synergistically induces the WIHN [19]. RA also induces hair-follicle regeneration and hair-follicle stem cell activation through Wnt signaling [34,44]. In addition, PF573288 and blebbistatin, small molecules that inhibit focal adhesion kinase (FAK) and myosin II, respectively, also significantly induce the WIHN [45]. Specifically, FAK inhibition is known to induce the phosphorylation of STAT3, which is induced by Wnt signaling and promotes WIHN in the wounds [45].

There have been many reports so far on the development of drugs that promote a hair-cycle transition [46,47], but these drugs are unlikely to be the ultimate treatment for alopecia lacking HFSCs [17,43]. Given the regenerative capacity of WIHN, drugs that promote WIHN are desirable but currently unavailable for alopecia treatment, but recent research into the underlying mechanisms of WIHN may ultimately enable the development of drugs that promote WIHN. Overall, developing drugs to increase the effectiveness of WIHN will provide treatment opportunities for many patients suffering from alopecia.

**Table 3:** Various drug candidates promoting the WIHN.

Substances	Types	Mechanisms	References
VPA	Small molecule	By inhibiting GSK-3β	[41]
PBA	Small molecule	By inhibiting GSK-3β	[41]
LiCl	Small molecule	By inhibiting GSK-3β	[29]
IL-6	Recombinant protein	By inducing expression of keratinocyte differentiation	[33]
PTD-DBM	Peptide	By inhibiting CXXC5-Dvl interaction	[17]
KY19382	Small molecule	By inhibiting CXXC5-Dvl interaction	[43]
TEPP-46	Small molecule	Through crosstalk with Wnt signaling	[19]
RA	Small molecule	By activating Wnt signaling	[34,44]
PF573288	Small molecule	By inhibiting FAK	[45]
Blebbistatin	Small molecule	By inhibiting myosin II	[45]

## 8. Conclusions and Perspectives

The WIHN is a regenerative phenomenon that can be utilized to treat alopecia [3]. The WIHN involves both developmental and adult wound-healing processes [7], and the WIHN is regulated by a variety of signaling pathways [3,8]. Specifically, WIHN involves both signaling associated with developmental processes, such as Wnt and SHH signaling, and signaling associated with the wound-healing process, such as TGFβ and IL-6 signaling [8,32,33]. The role of multiple signaling pathways in WIHN has been validated by several transgenic mice, and these studies have contributed to the efficient induction of WIHN by the activation of molecular signaling pathways [3,17].

Clinical application of WIHN has been difficult due to the lack of dermal γδ T cells in human scalps [30], but drug development including Wnt signaling activators is increasing the effectiveness of WIHN in humans [17,43]. Translational studies using various animal models have also made important contributions to the development of potential drugs to induce WIHN in humans [48]. Importantly, the development of these drugs for practical and clinical purposes may provide many opportunities to treat alopecia. Moreover, given the relationship between scar formation and the WIHN, further studies on the WIHN may offer unprecedented opportunities to treat scarring diseases such as cicatricial alopecia and burns [49,50]. In conclusion, understanding the underlying mechanisms that regulate the WIHN may be beneficial for the treatment of skin disorders, including hair loss.

## Figures and Tables

**Table 1:** The key molecular signaling pathways involved in the WIHN.

Signaling (or Its Component)	Mechanisms	References
Wnt signaling	By activating β-catenin, the downstream effector of this signaling	[3]
SHH signaling	By activating GLI1, the downstream effector of this signaling	[8]
IGF1 signaling	Through the contribution of EGFR-positive mesenchymal cells	[13]
BMP signaling	By increasing Msx2, the downstream target of this signaling	[14]
HIF-1α signaling	By stimulating glutamine metabolism	[15]

**Table 2:** Multiple signaling pathways in immune cells and non-immune cells during the WIHN.

Signaling	Cellular Sources	Mechanisms	References
FGF9 signaling	γδ T cells	By activating Wnt signaling	[30]
TLR9 signaling	γδ T cells	By up-regulating AREG expression	[31]
TNF-α signaling	Macrophages	By activating AKT/β-catenin signaling	[25]
CX3CR1 signaling	Macrophages	By up-regulating TNF-α expression	[32]
TLR3 signaling	Keratinocytes	By inducing IL-6 expression and STAT3 phosphorylation	[33]
RA signaling	Keratinocytes	By inducing IL-6 expression	[34]
HIF-1α signaling	Keratinocytes	By inducing IL-1β production	[15]
IL36 α signaling	Keratinocytes	By activating the IL-6/STAT3 pathway	[35]

## Data Availability

The data presented in this study are available on request from the corresponding author.
